# Cyclosporine reduces the spleen dimensions in rabbits

**DOI:** 10.1590/ACB360402

**Published:** 2021-05-07

**Authors:** Luiz Ronaldo Alberti, Leonardo de Souza Vasconcellos, Andy Petroianu

**Affiliations:** 1 Associate Professor. Universidade Federal de Minas Gerais – Medical School – Department of Surgery – Belo Horizonte (MG), Brazil.; 2 Associate Professor. Universidade Federal de Minas Gerais – Medical School – Department of Propedeutics – Belo Horizonte (MG), Brazil.; 3 Full Professor. Universidade Federal de Minas Gerais – Medical School – Department of Surgery – Belo Horizonte (MG), Brazil.

**Keywords:** Spleen, Cyclosporine, Morphology, Histology, Immunology, Rabbits

## Abstract

**Purpose:**

To assess the influence of prolonged cyclosporine use on the macro- and microscopic morphology of the spleen.

**Methods:**

16 adult rabbits were divided into two groups (n = 8): group 1 – a placebo group, which was followed-up over a period of nine months; group 2 – which had taken an oral dose of cyclosporine (10 mg·kg^–1^·day^–1^) over nine months. At the end of this period, the splenic histoarchitecture of all animals was evaluated and the splenic corpuscles were measured.

**Results:**

The spleens of the first group presented normal characteristics and dimensions. The second group, however, had a reduction in all dimensions and its tissue texture had become soft. The white pulp and the perivascular sheath had become reduced in size and the number of lymphoid follicles had also fallen (p = 0.002), manifesting less splenic corpuscles (p = 0.0012) and lymphocyte nuclear pigments (p = 0.03).

**Conclusions:**

Prolonged use of cyclosporine reduces the spleen size, transforming it into a soft organ associated with a decrease in white pulp, perivascular sheath, lymphoid follicles and nuclear pigments in rabbits.

## Introduction

The spleen has relevant immune functions, including the removal of antigens from the bloodstream and the production of antibodies, immunoglobulins, amino acids, lymphocytes, monocytes and opsonins. It is also particularly important for complement factors, fibronectin, C-reactive protein, tuftsin and properdin^1^-^4^.

Cyclosporine has an immunosuppressive effect based on cellular immune response and causes a reduction of antibody-dependent T lymphocytes^5^. In terms of side effects, this drug interferes with the endocrine system, causing changes in gonadal function and prolactin levels, decreasing fertility and fetal development^5^-^7^. The drug is also nephrotoxic and is associated with systemic arterial hypertension, neurotoxicity and sepsis. Some clinical disorders, such as nausea, vomiting, diarrhea and abdominal discomfort, have also been frequently described^7^-^12^.

However, the side effects of cyclosporine have not been entirely understood in relation to organs like the spleen, which have not been studied after exposure to immunosuppressants. The purpose of this study was to assess the influence of prolonged cyclosporine use on the macro- and microscopic morphology of the spleen.

## Methods

The study has been conducted at the Vivarium of the Medical School from the Universidade Federal de Minas Gerais (UFMG) after approval from the Animal Research Ethics Committee (CEUA/UFMG), protocol 185/12.

Sixteen male rabbits (*Oryctolagus cuniculus*) with an initial weight between 2500 and 3000 g were kept in individual cages and received water and feed *ad libitum*. The animals were randomly divided into two groups (n = 8):

Group 1 – The placebo group, which was followed-up over nine months.

Group 2 – Was given a daily dose of cyclosporine(10 mg**·**kg^–1^ **·**day^–1^) diluted in 10 mL of milk, administered through an orogastric catheter (12 Fr), over a nine-month period.

At the end of the follow-up period, under intravenous anesthesia using ketamine (25 mg**·**kg^–1^), all animals from the two groups were submitted to a median laparotomy and the spleen was removed for macroscopic assessment. Its longitudinal and transverse dimensions were measured, the spleen volume was calculated, and the consistency was assessed.

The splenic tissue was soaked in Bouin’s solution and processed for histological examination. Five µm thick sections were mounted and stained with hematoxylin and eosin, whereas Masson’s trichrome technique was applied in order to examine the collagen. The splenic histoarchitecture was measured with a micrometer at intervals of 1000 µm, the histological assessment was carried out under light microscopy for morphometric analysis. The diameters of 30 splenic corpuscles and their germinal centers were measured and their area (µm^2^) and perimeter (µm) calculated.

The results were compared using the chi-squared test and Student’s t test. The differences were considered significant for values corresponding to p < 0.05.

## Results

All animals survived during the nine months of the experiment without complications. Their weight increased uniformly and the amount of cyclosporine administered to group 2 was weekly adjusted according to the weight of each rabbit.

The mean dimensions of the spleens in group 2 were lower and the tissue was much softer than those from group 1 (p = 0.002) (Table 1).

**Table 1:** Values of mean ± standard error of mean of spleens and their white pulp corpuscles.

Variables	Group 1	Group 2	p
Dimensions (L × T) (cm)	6.12 ± 0.9 × 2.23 ± 0.45	4.53 ± 0.8 × 1.85 ± 0.37	0.002
Area (µm^2^)	234.519.34 ± 4.150.73	132.853.72 ± 2.454.94	0.0012
Maximum diameter (µm)	702.53 ± 14.19	450.55 ± 85.99	0.003
Minimum diameter (µm)	552.32 ± 39.41	310.69 ± 25.86	0.004
Perimeter (µm)	2193.62 ± 112.05	1234.59 ± 95.05	0.001

Group 1: normal rabbits; without medication followed-up over nine months. Group 2: rabbits after using cyclosporine (10 mg·kg^–1^·day^–1^) followed-up over nine months. L – longitudinal size, T – transverse size.

**Figure 1:**
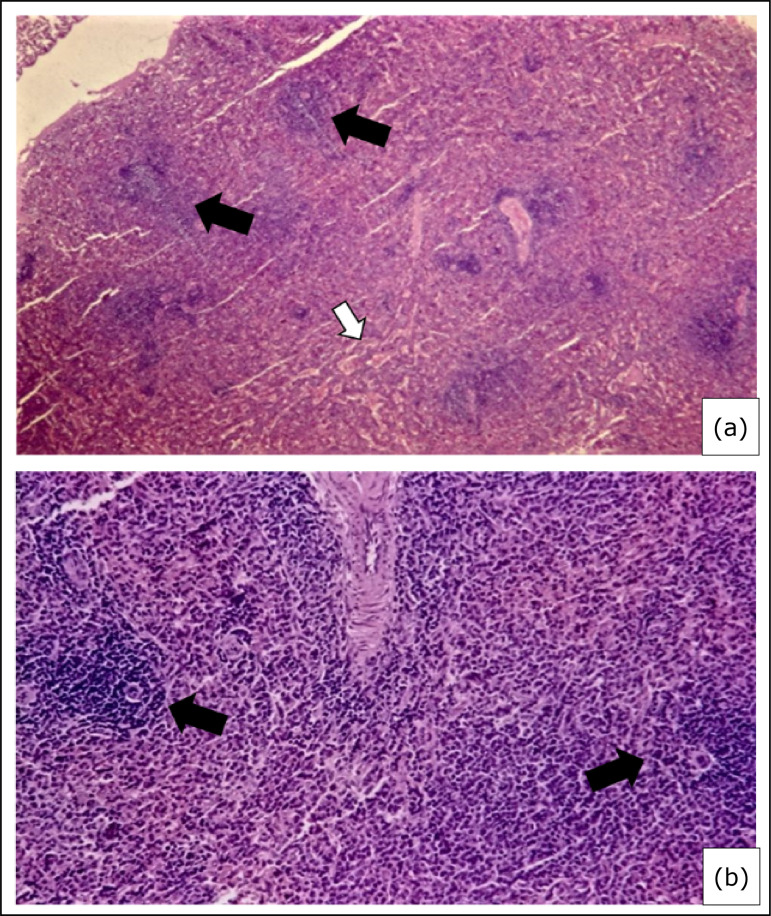
Photomicrograph of splenic parenchyma in a group 1 rabbit (without having taken cyclosporine). **(a)** Observe the normal aspect of red (white arrow) and white (black arrows) pulps; **(b)** Normal vascularity numerous lymphoid follicles (arrows) at different stages of maturation as well as a perivascular sheath manifesting an abnormal size (Hematoxylin and Eosin, 400×).

**Figure 2:**
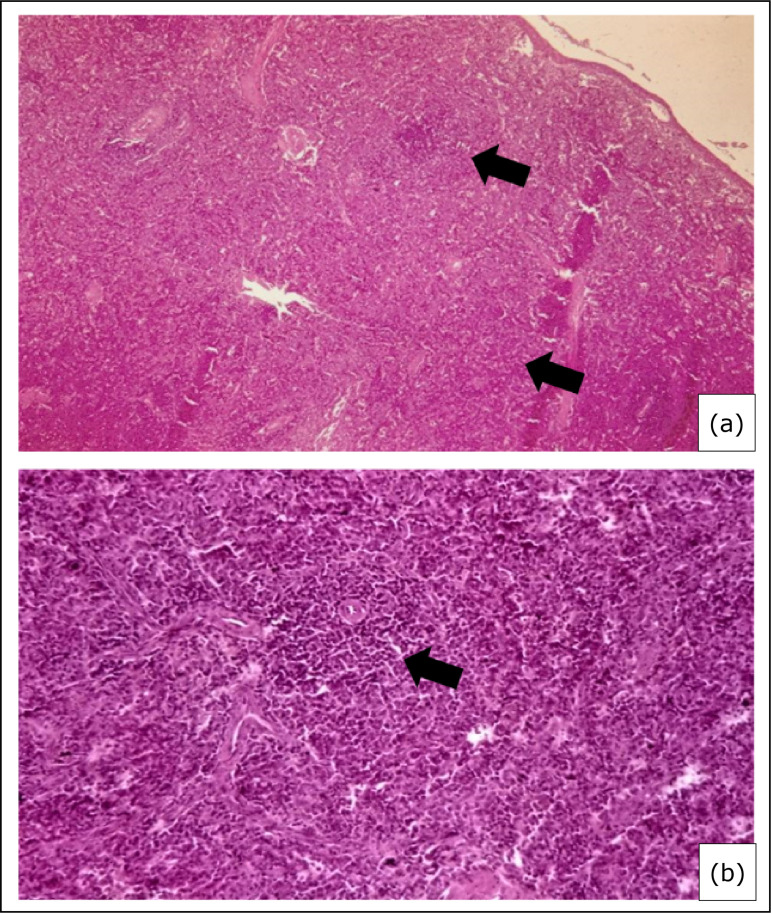
Photomicrograph of an aplastic splenic parenchyma of a group 2 rabbit after nine months use of cyclosporine (10 mg·kg^–1^·day^–1^). **(a)** Observe reduction in the white pulp (arrows); **(b)** Reduced cellularity in the lymphoid follicles of the white pulp (arrow) (Hematoxylin and Eosin, 400×).

The splenic parenchyma of all rabbits from group 1 presented a normal white and red pulp, with numerous lymphoid follicles at different stages of maturation, as well as perivascular sheaths of varying size (Fig. 1). On the other hand, the spleens taken from group 2 were significantly reduced in size and exhibited a white pulp, lymphoid follicles, perivascular sheath and nuclear lymphocyte pigmentation (Fig. 2). No signs of inflammation, ischemia or necrosis were identified in the spleens of either group.

The morphometric results of the measurements made on the splenic corpuscles and the germinal centers of the white pulp are shown in Table 1. There was a reduction in the splenic corpuscles in group 2 compared to group 1 (p = 0.03). All dimensions of splenic corpuscles in group 2 were smaller than those in group 1: area (p = 0.0012), maximum diameter (p = 0.003), minimum diameter(p = 0.004) and perimeter (p = 0.001).

## Discussion

This experimental study aimed to investigatethe effects of the prolonged use of cyclosporine on thespleen morphology. After nine months, there was a substantial reduction in the splenic size, its consistency and these macroscopic parameters were associated with a significant decrease in lymphoid follicle cellularity and perivascular sheaths. These results are in accordance with other studies, which have shown the inhibitory effect of cyclosporine on T lymphocyte activation and proliferation associated with the cytoplasmic heterodimeric complex receptor^8^-^11^. This reduces the movement of the nuclear factor of activated T lymphocytes and impairs cytokine gene induction, principally interleukins 2 and 4, as well as tumor necrosis factors α and γ^12^-^15^.

The prolonged use of cyclosporine A did not cause inflammation, damage, necrosis or vascular alteration in the spleens^16^. On the other hand, the drug was associated with a reduction of 43% in the white pulp, without manifesting any morphological disorder in the red pulp. These results differ from the studies carried by Armas *et al*.^9^, who did not identify any splenic change in rats submitted to prolonged use of cyclosporine. On the other hand, in a study with 24,939 transplanted patients who had been using cyclosporine for more than a year, 110 (0.45%) of them showed splenomegaly proportionally higher in males and elders in 61% of these records. However, there is no record of the action of cyclosporine in the other 99.55% of patients in this group^14^.

Pharmacodynamic studies have investigated ultramicronized cyclosporine (atopic) in normal animals. An initial *in vitro* investigation demonstrated cyclosporine-mediated suppression of T-lymphocyte activation-related molecules and cytokines^16^. Peripheral blood mononuclear cells were isolated and activated, with half of the cells incubated while exposed to cyclosporine, and the other half not exposed to the drug. Cells were then analyzed using flow cytometry, with T-cell expression of the intracellular cytokines interleukin- 2 (IL)-2, IL-4, and interferon (IFN-c) evaluated after drug exposure. All cytokines demonstrated a time-dependent suppression profile. The T-cell surface molecules CD25 and CD95, which have roles in T-cell activation and development, were evaluated after drug exposure, and there was also significant suppression of expression of both biomarkers in the presence of cyclosporine. In a subsequent *in vivo* study, activated T-cell expression of IL-2, IL-4, and IFN-c was investigated by flow cytometry when animals were treated with two different oral cyclosporine dosages. With high-dose cyclosporine, activated T-cell expression of IL-2 and IFN-c was significantly suppressed, but IL-4 was not similarly affected. Even with this low dosage of cyclosporine, however, T-cell expression of IFN-c was still significantly suppressed. Mean T-cell expression of IL-2 also was decreased^16^.

No studies have focused on the effect of cyclosporine on human spleens, which must be investigated using imaging methods and studying autopsies of patients who have undergone transplants. Other immunosuppressants should be studied in order to verify their impact on the spleen morphology and function.

## Conclusion

The prolonged use of cyclosporine in rabbits softens and reduces the size of the spleen, is associated with a decrease in white pulp, the perivascular sheath, lymphoid follicles and nuclear pigmentation.
